# Rare Transverse Vaginal Septum in an Adolescent Girl

**DOI:** 10.7759/cureus.31623

**Published:** 2022-11-17

**Authors:** Poojan Dogra, Neha Chauhan, Mridul Soni

**Affiliations:** 1 Obstetrics and Gynaecology, All India Institute of Medical Sciences, Bilaspur, IND; 2 Obstetrics and Gynaecology, Shri Lal Bahadur Shastri Government Medical College, Mandi, IND; 3 Research, Shri Lal Bahadur Shastri Government Medical College, Mandi, IND

**Keywords:** laparoscopic technique, ultrasonography, female genital tract, haematocolpos, transverse vaginal septum

## Abstract

An uncommon malformation of the female genital system is the transverse septum which is often known as the vaginal septum. A problem with the union and channelling of the Mullerian conductors and urogenital sinus is the primary common cause of progressive pathogenesis. This anatomical blockage can block the vagina, which can lead to a hematocolpos that is connected with periodic pelvic discomfort in teenage females immediately after menarche. A thorough clinical gynaecological evaluation, particularly an abdominal or transrectal ultrasound scan, and in even more complicated situations, magnetic resonance imaging is used to determine the presence of a vaginal septum. The surgical intervention must be performed as soon as feasible. We report the management of a case of a 16-year-old girl presented with primary amenorrhea associated with severe pain in the lower abdomen for 2-3 days, which was cyclic in nature and spasmodic in character. She revealed a hematocolpos during the examination, aggravating a full transverse vaginal septum. Removing the hematocolpos and examining the cervix were the steps in the therapy.

## Introduction

The treatment of congenital defects is a minor yet significant aspect of the work of a gynaecologist. A transverse vaginal septum is a subset of these abnormalities, and it differs from the more prevalent longitudinal deformities subgroup in terms of its genesis, occurrence, clinical presentation, and prognosis. The literature reports initially found information conducted in 1979, which suggested that the event ranges for it was from 1:2100 to 1:84000 [1]. The total or partial restriction of vaginal flow is typically connected to the pathological features of such septa. The individual will report dyspareunia and dysmenorrhea with an imperfect septum that permits incomplete outflow. A defect in the fusion of the urogenital sinus and Mullerian ducts is responsible for this. About 72% of the transverse septum is found in the lower part of the vagina, 22% in the central part, and only 6% in the upper one-third of the vagina [2-3]. The majority of these patients receive successful treatment through surgical septum repair surgery. It is challenging to treat the transverse septum due to the very high chances of subsequent vaginal stenosis, especially in an adolescent girl, as discussed in this case study.

## Case presentation

**Figure 1:**
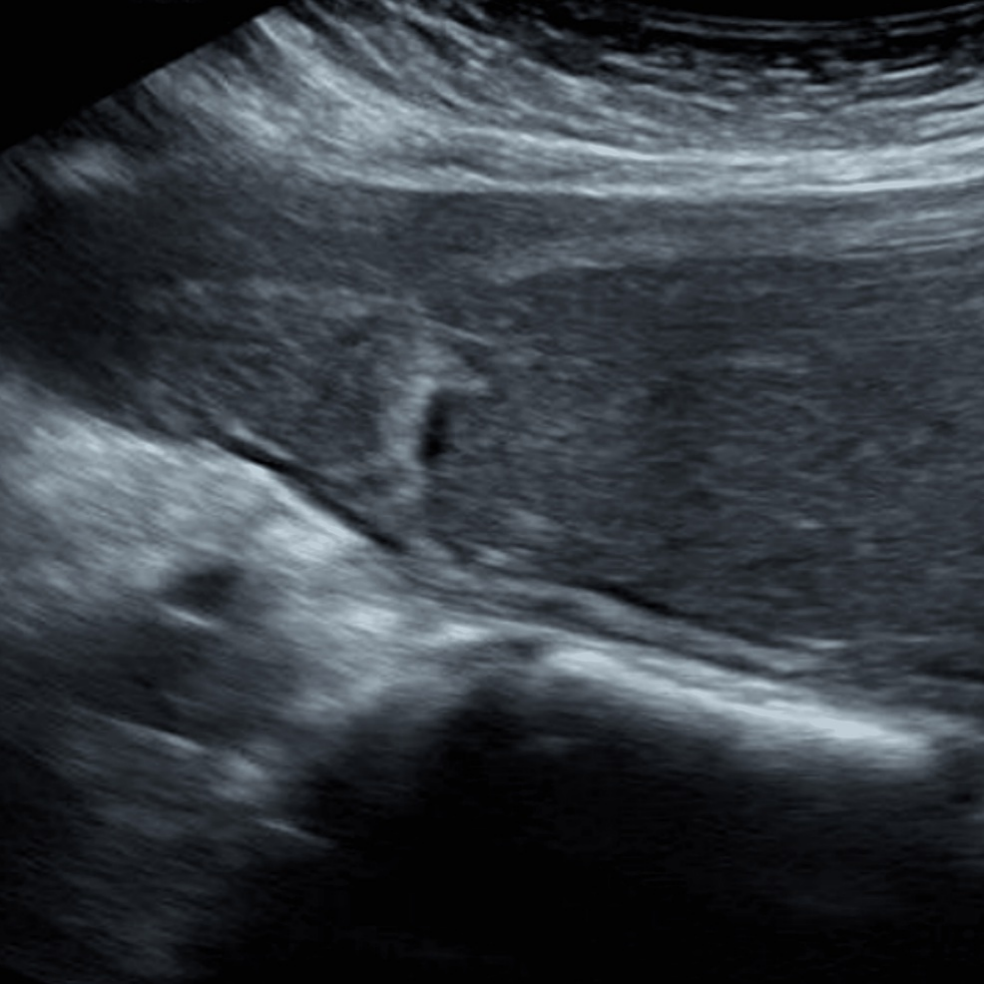
Ultrasonography revealed hematocolpos

A 16-year-old girl presented with primary amenorrhea associated with severe pain in the lower abdomen for 2 to 3 days, which was cyclic in nature and spasmodic in character. On examination, her general condition and vitals were stable. Fully developed secondary sexual characters were observed. The hymen was intact, with a central opening. No bulge on supra-pubic pressure. Ultrasonography was done, which revealed hematocolpos, as shown in Figure 1.

**Figure 2:**
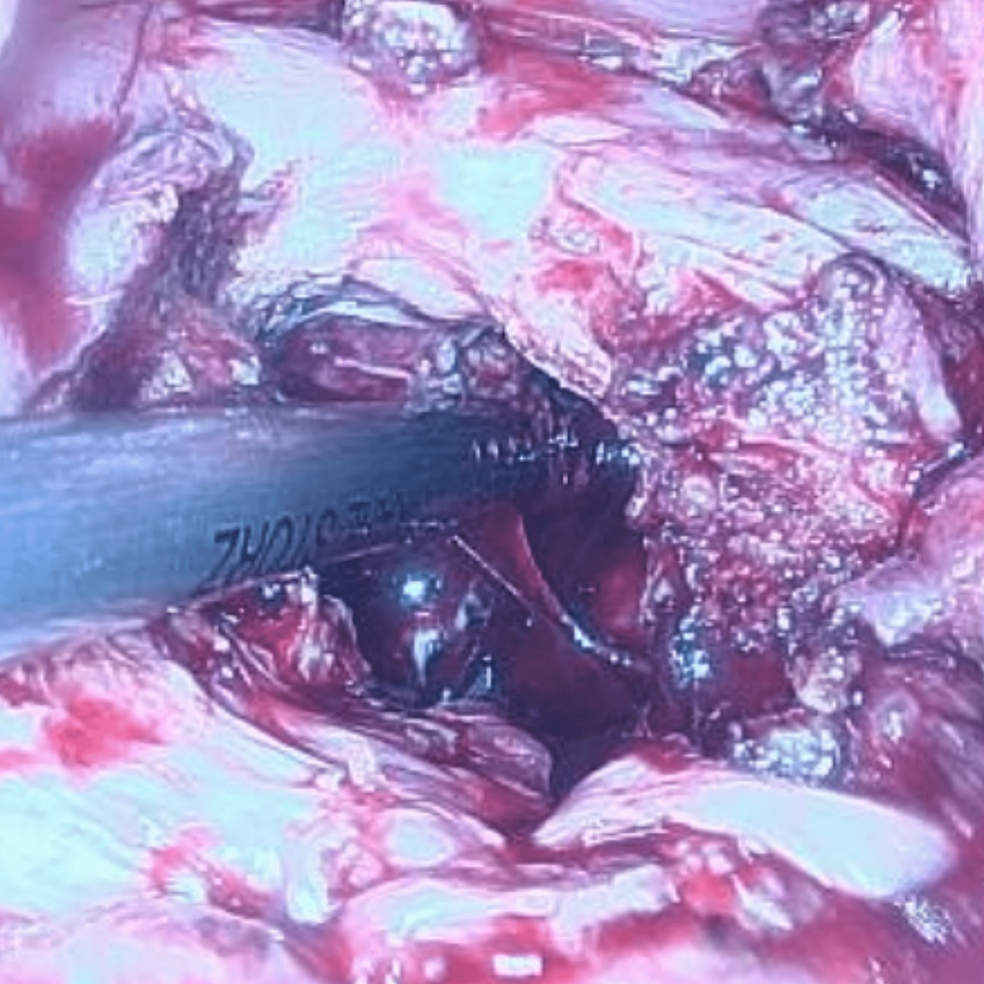
Laparoscopic image of the hysterectomy

On magnetic resonance imaging, a thick transverse vaginal septum was noticed in the upper one-third of the vagina. On examination under anesthesia, the transverse septum was identified high up, and vaginal resection of the septum was done. Intra-operatively, the rectal injury occurred, which was corrected by a surgeon. The patient was discharged in stable condition on the seventh postoperative day. After two-and-half months, the patient again presented with similar complaints of pain in the lower abdomen and amenorrhea. On ultrasonography, the hematometra and hematocolpos were observed. A repeat magnetic resonance imaging revealed a 3 to 4 centimeter thick septum in the upper one-third of the vagina with a stenosed (fibrosis in the passage) vagina. So, this time patient had restenosis of the septum and fibrosed passage of the vagina. Therefore, an abdominoperineal approach to excise the septum and creation of the neo-vagina was chosen. The transverse septum was resected through a hysterectomy, done laparoscopically. The stenosed vagina was recreated vaginally. The intracervical catheter inflated at the level of the excised transverse septum and was kept for two weeks. A vaginal mold with estrogen cream was kept to prevent restenosis. The patient was followed up in the post-operative period and was stable with regular menses. Laparoscopic image of the hysterectomy that was done on the patient followed by the resection of the transverse septum as shown in Figure 2.

## Discussion

The vaginal septum was first described in 1877; since then, other case series have been published. However, they remain few due to the rarity of the disease. During pre-puberty, the patient may present with hydrocolpos caused by an obstruction in the drainage of genital secretion caused by hypersecretion of the proximal reproductive glands in response to the maternal hormonal response. In the post-pubertal period, the presentation will depend on whether the septum is complete or perforated. A complete septum will present with non-specific symptoms like pain in the lower abdomen, pain in the lower back, constipation, or urinary retention associated with primary amenorrhea, while a perforated septum will generally present with dyspareunia and dysmenorrhea. There are various treatments and techniques which are available. Dennie et al., proposed that the septum should be resected once the girl reaches the age of first menstruation, and results are good if treatment includes drainage of hematocolpos [4]. Three routes are usually involved. First is the laparotomy abdominoperineal route vaginoplasty, second is the simple vaginal resection, third is the laparoscopic vaginal resection. The Grunberger technique involves a cross incision in the caudal part and a cross incision in the other part with transverse closure. Vaginal stenosis remains the most common complication. Postoperative vaginal dilatation may help reduce the scarring and stenosis of the vaginal septum. Management of the vaginal septum with drainage of hematocolpos is necessary to preserve fertility and prevent chances of developing endometriosis [5].

## Conclusions

The transverse vaginal septum is a rare Mullerian anomaly. Early diagnosis and surgical treatment with drainage of hematocolpos remain the mainstay of treatment. Restenosis, infertility, and endometriosis are well-known complications of the disease. This literature research backs up the neutral qualities and positive prognosis following surgical therapy. The notion that the transverse septum results from inadequate canalization of such vaginal plate are supported by the documented pathophysiology of the septa. To resolve the debate about the vaginal plate's genesis, more study is required.
